# Toward *Mycobacterium tuberculosis* Virulence Inhibition: Beyond Cell Wall

**DOI:** 10.3390/microorganisms13010021

**Published:** 2024-12-26

**Authors:** Maria S. Kotliarova, Mikhail S. Shumkov, Anna V. Goncharenko

**Affiliations:** Bach Institute of Biochemistry, Fundamentals of Biotechnology, Federal Research Center, Russian Academy of Sciences, Moscow 119071, Russia; shumkovm@gmail.com (M.S.S.); pylaevanna@gmail.com (A.V.G.)

**Keywords:** virulence, anti-virulence drugs, tuberculosis

## Abstract

*Mycobacterium tuberculosis* (Mtb) is one of the most successful bacterial pathogens in human history. Even in the antibiotic era, Mtb is widespread and causes millions of new cases of tuberculosis each year. The ability to disrupt the host’s innate and adaptive immunity, as well as natural persistence, complicates disease control. Tuberculosis traditional therapy involves the long-term use of several antibiotics. Treatment failures are often associated with the development of resistance to one or more drugs. The development of medicines that act on new targets will expand treatment options for tuberculosis caused by multidrug-resistant or extensively drug-resistant Mtb. Therefore, the development of drugs that target virulence factors is an attractive strategy. Such medicines do not have a direct bacteriostatic or bactericidal effect, but can disarm the pathogen so that the host immune system becomes able to eliminate it. Although cell wall-associated targets are being actively studied for anti-TB drug development, other virulence factors important for adaptation and host interaction are also worth comprehensive analysis. In this review, specific Mtb virulence factors (such as secreted phosphatases, regulatory systems, and the ESX-1 secretion system) are identified as promising targets for novel anti-virulence drug development. Additionally, models for the search of virulence inhibitors are discussed, such as virtual screening in silico, in vitro enzyme inhibition assay, the use of recombinant Mtb strains with reporter constructs, phenotypic analysis using in vitro cell infection models and specific environments.

## 1. Introduction

About a quarter of the world’s human population is latently infected with *Mycobacterium tuberculosis* (Mtb). About 5–10% of latent infection cases eventually progress to active disease. In addition, tuberculosis (TB) remains one of the deadliest infectious diseases. According to WHO, about 1.3 million people died due to the disease in 2022 [1]. The thick, complexly arranged hydrophobic cell wall of Mtb [2] and its ability to persist for long periods of time in an inactive dormant state [3] are the factors that complicate Mtb treatment and contribute to the development of pathogen resistance to antibacterials. Current Mtb treatment regimens include three or four drugs for 6–9 months, and usually under direct medical observation [4,5].

Prolonged courses and possible violations of the therapy regimens lead to the development of pathogen resistance [6,7]. Of particular concern is the emergence and spreading of multidrug-resistant and extensively drug-resistant Mtb strains. Therefore, the search for new compounds and targets for the treatment of TB is necessary. Drugs with a new mechanism of action could greatly expand the possibilities for controlling drug-resistant tuberculosis. In this regard, new targets have been identified recently, and several promising compounds are under clinical trial [8,9,10,11]. However, the problem of finding new, more effective regimens for the treatment of tuberculosis is far from being solved and the search is still ongoing.

The difficulties are primarily connected with the appearance and spread of resistant bacteria, that become insusceptible to the new drugs. Resistance to the most recently approved for use antibiotics, bedaquiline and delamanid (included in WHO recommended treatment regimens in just 2013 and 2014, respectively), is already detected in multidrug-resistant Mtb isolates [12]. According to a systematic review, the phenotypic- and genotypic-acquired resistance constituted 2.2% (1.1–4.6%) and 4.4% (1.8–5.8%), respectively [13]. Thus, the search for drugs that would provide a slowed rate of resistance development is of particular relevance.

A promising strategy for the development of new drugs that will overcome the problem of antibiotic resistance emergence is the search for anti-virulence drugs [14,15]. This idea implies the use of drugs that inhibit virulence factors and consequently the mechanisms of infection establishment, allowing the host immune system to eliminate the pathogen [16,17]. Since virulence inhibitors do not affect the viability and growth capacity of bacteria directly, selective survival and resistance development rates are expected to be reduced compared to classical antibiotics.

Thus, the development of effective virulence inhibitors is a fundamentally new approach that promises to be an important step towards the solving of the problem of global antibiotic resistance of clinically important pathogens. To date, the greatest success in the implementation of the approach has been achieved in the case of antibodies to bacterial exotoxins (several antibody preparations have been approved for clinical use against the toxins of *Clostridium botulinum*, *Bacillus anthracis* and *Clostridium difficile*) [17]. Small molecule-based drugs are being developed that target exotoxins, adhesion and biofilm formation factors, effectors interacting with the immune system and their secretion systems, factors involved in the pathogen adaptation to stress, quorum sensing factors and regulatory systems. However, the path to the development of drugs of this type seems to be quite complicated.

For virulence inhibitors, there are no well-defined and standardized models for determining their activity and estimating the required dose. This is due to the specificity of the drugs that lack a direct bactericidal or bacteriostatic action allowing an estimation of a minimum inhibitory concentration. Nevertheless, at least in animal models, evidence has been obtained that such substances could be effective [18].

According to the virulence factor database (VFDB) (VFDB, http://www.mgc.ac.cn/VFs/, accessed on 1 December 2024) [19], 902 anti-virulence compounds of 17 superclasses targeting 32 bacterial genera are in various stages of preclinical development now and only four compounds have reached the stage of clinical trials. Through the extensive literature mining, the database has integrated information on the virulence factors of bacterial pathogens and also systematically collected public data on anti-virulence compounds.

This review is devoted to the comprehensive analysis of the problem of anti-virulence drug development. In the first part of it, we are going to briefly discuss the pathogenesis of Mtb and the virulence factors that ensure its success in infection establishment. The second part will highlight the models which could be used for screening studies to find active compounds. Finally, the examples of compounds will be discussed that have been shown to be biologically active in terms of the inhibition of mycobacterial virulence factors that enable pathogen–host interactions. This review is mainly focused on protein virulence factors that disrupt host immune functions. We will tackle the systems that ensure the expression of the proteins and their delivery to cells as well.

## 2. Pathogenesis of Tuberculosis and Mtb Virulence Factors

The high prevalence of Mtb in the human population, as well as the complexity of tuberculosis treatment, are the consequences of the pathogen’s distinctive features, including its ability to adapt to an intracellular environment, modulate host immune responses and persist for extended periods of time in a dormant unreplicated state [20,21,22].

**Figure 1:**
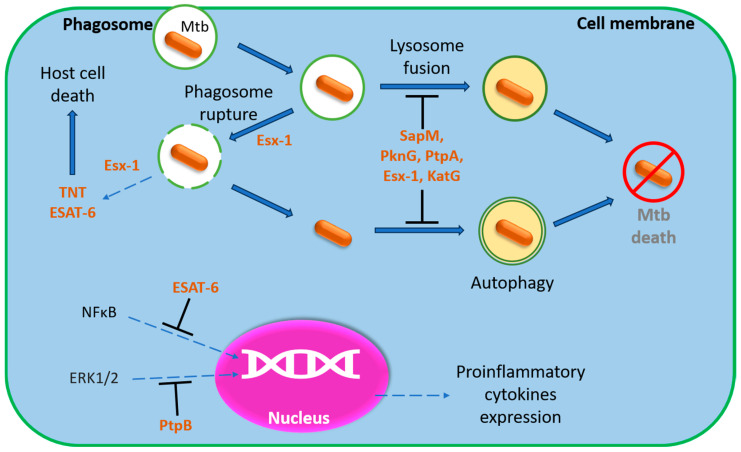
Schematic representation of mycobacterium tuberculosis influence on the key components of the phagocyte antimicrobial response. Details are given in the text.

The primary pathogen strategy in the initial stages of the disease is the bacteria intracellular persistence and multiplication in infected cells, including professional phagocytes such as macrophages and dendritic cells [23]. For this purpose, the bacterium has evolved multiple adaptations to survive within host cells and subvert the host immune response (Figure 1). The main mechanisms include delayed phagosome maturation, phagosome rupture, suppression and subversion of host immune responses and the lytic death of host cells that facilitate pathogen dissemination.

The bacterium engulfed by the macrophage enters the phagosome, which matures through a series of fusions with early and late endosomes, and eventually with the lysosome [24]. As the phagosome matures, it acidifies and acquires bactericidal activity, provided by reactive oxygen and nitrogen species, antimicrobial peptides and specific proteases and hydrolases [25]. In addition to the elimination of pathogens, these processes are crucial for the processing and presentation of antigenic peptides by MHCII [26] and, probably, for the cross-presentation by MHC I [27], which is essential for the development of adaptive immunity.

Specifically, the processes of endosome fusions necessitate the coordinated involvement of a multiple molecular factors including phosphoinositide 3-kinases, Rab GTPase and its effectors, tethering factors, vacuolar sorting proteins (Vps), soluble N-ethylmaleimide-sensitive fusion factor attachment proteins receptors and N-ethylmaleimide-sensitive factor [28]. Mtb inhibits the maturation process of the phagosome at the stage of the early endosome [29,30]. Delayed maturation is associated with cell wall components such as lipoarabinomannan [31], glycolipid trehalose-6,6′-dimycolate [32] and some secreted proteins like secreted acid phosphatase (SapM) [33], nucleoside diphosphate kinase [34], protein tyrosine phosphatase A (PtpA) [35] and protein kinase G (PknG) [36].

Mtb survival in phagosomes and phagolysosomes is determined by the ability of bacteria to adapt their metabolism to the intracellular environment and evade antimicrobial mechanisms of eucaryotic cells, in particular, reactive oxygen species (ROS) and reactive nitrogen species. The defense of Mtb against peroxides generated by phagocyte NADPH-oxidase is performed by the catalase-peroxidase KatG [37].

The other ROS, produced by macrophages, are superoxide radicals. Bacterial cells protect themselves from the ROS by scavenging superoxide radicals with the superoxide dismutases, SODA and SODC [38,39]. These proteins have been demonstrated to significantly reduce NO radicals and ROS production and impair immunological cell functions in the early stages of TB infection [40].

When Mtb faces iron deficiency in the phagosome, it actively expresses the siderophores, carboxymycobactins and mycobactins. The latter chelate iron ions from the host environment and transport them into the bacterial cell. From here, targeting the siderophores seems to be a viable approach to inhibit the virulence of the bacterium in the host [41]. The important role of mycobactins as a virulence factor in the course of TB infection has been confirmed by the genetic disruption of the *mbtB* gene, that results in the inhibition of mycobactin production and slowing down the Mtb growth rate [42].

The next crucial event in the intracellular pathogenesis of Mtb is the phagosome rupture. Its primary mechanism involves the ESX-1 secretion system and its effectors, as well as phthiocerol dimycocerosate [43,44]. Following the rupture, Mtb components enter the host cell cytoplasm, leading to the disruption of various immune response mechanisms. In particular, the entry of mycobacterial DNA into the cytoplasm of host cells leads to activation of cytosolic DNA sensor, cyclic GMP-AMP synthase (cGAS), and subsequent activation of type I interferon (IFN) production via stimulator of interferon genes (STING) [45].

The role of type I IFN signaling in tuberculosis infection is controversial. On the one hand, deletion of type I IFN receptor increases the resistance of TB-susceptible mice [46] and the type I IFN-inducible signature in patients’ blood has been associated with TB progression in humans [47]. On the other hand, the STING pathway also leads to selective autophagy of *M. tuberculosis* and the cytosolic release of the bacterial cyclic dinucleotide c-di-AMP. In addition, Mtb has been shown to secrete the bacterial phosphodiesterase CdnP, which cleaves cyclic dinucleotides and prevents their recognition and subsequent activation of STING and the type I IFN response. In addition, by degrading c-di-AMP and c-di-GMP into nonimmunogenic nucleotides, CdnP reduces the immune detection of these bacterial-derived compounds [48].

Autophagy is a process by which intracellular macromolecules, organelles or invading pathogens undergo a lysosomal-dependent degradation process. The process is important for maintaining homeostasis and is also a protective antimicrobial mechanism [49].

Mtb has multiple mechanisms to inhibit this process, including the secretion of SapM [50] and PtpA [51]. PknG can induce autophagy but also inhibit autophagosome maturation [52]. The ESX-1 secretion system is also required for this inhibition [53]. However, starvation- or cytokine-induced autophagy has been shown to overcome phagosome–lysosome fusion arrest. This has been shown to result in the elimination of the Mtb strain H37Rv while the Beijing strain was resistant to autophagy probably due to KatG upregulation [54].

The EsxA effector (also known as ESAT-6 protein) is secreted as a 1:1 heterodimer with EsxB (CFP-10) and is essential for the phagosome rupture and the subsequent Mtb release into the cytoplasm of macrophages. The uncoupling of ESAT-6 from CFP-10 and its activity are induced by acidification [55]. In addition, ESAT-6 has additional effector functions that are important for virulence, including the suppression of innate and adaptive immunity signaling [56,57]. Thus, the protein induces apoptosis in human macrophages by activating the expression of caspases. It has been shown that infection of wild-type macrophage cells with Mtb strain H37Rv resulted in significant apoptosis, whereas a deletion mutant that does not express ESAT-6 did not exert such an effect. The hypothesis was put forward that the membrane pore formation may be the primary mechanism by which ESAT-6 induces an apoptotic response [58].

A study on the Mtb-related fish pathogen *M. marinum* revealed that ESAT-6 is also associated with mitochondrial membrane damage, which can lead to lytic cell death, and the target of rapamycin mTOR, a master regulator of metabolism, is preventing this process. Impairment of mTOR function was associated with necrotic cell death and granuloma progression [59].

The functional ESX-1 system is strictly essential for the lysis of fibroblasts and macrophages in in vitro infection model [60]. Recently tuberculosis necrotizing toxin (TNT) was identified [61]. It is the C-terminal part of the channel-forming necrosis-inducing toxin (CpnT), which can be detached and secreted into the external environment [62]. Although toxin secretion is provided by CpnT, access of TNT to the cytosol is entirely dependent on the ESX-1 induced phagosome membrane damage [63].

Intracellular multiplication of Mtb and subsequent death of host cells leads to the spread of infection to newly arrived cells at the site of infection [14]. On the one hand, infected macrophages and dendritic cells represent a reservoir for infection. On the other hand, they activate the adaptive immune response by migrating to lymph nodes and priming naive T lymphocytes [64]. Mtb uses various mechanisms to disrupt these processes and delay the formation of the adaptive immune response [65]. When effector T cells eventually arrive at the site of infection, they activate macrophages via cytokines, primarily interferon gamma (INF-γ), which leads to the enhanced bactericidal action of macrophages. However, complete eradication of the pathogen is usually not achieved and a granuloma is formed at the site of infection. It is a dense, ordered structure composed of bacteria, infected macrophages and other cell types, including neutrophils, dendritic cells, B- and T-cells, natural killer cells and fibroblasts [66]. In the granuloma, Mtb is exposed to unfavorable conditions such as hypoxia, oxidative and nitrosative stress and broad nutrient starvation. However, mycobacteria are able to survive under these conditions for long periods of time. They enter a dormant state and persist until suitable conditions for reactivation occur [67,68].

In general, the ability to survive stressful conditions in metabolic quiescence is one of the determining mechanisms of Mtb success. The formation of metabolic quiescence (dormancy) is to a significant extent based on DosR regulon activity, which controls the response of Mtb to hypoxia, a key feature of the environment when the bacterium persists in a granuloma. DosR is activated by the kinase proteins, DosS and DosT [69,70]. Studies have shown that DosR mutants retain the ability to replicate but cannot persist and cause disease. In contrast, wild-type Mtb and the complemented mutants were able to develop infection. Lungs of animals infected with the mutants (but not the complementary strain) showed early transcriptional signs of T-cell recruitment, activation and proliferation, associated with an increase in the number of T cells that express homing and proliferation markers. Thus, DosR regulon modulates both the magnitude and the timing of adaptive immunity reactions in response to hypoxia in vivo that results in bacterial persistence [71]. Therefore, key components of the regulon can be considered as promising targets for the development of anti-virulence drugs.

As infection progresses and the immune response develops, Mtb must sense and respond to changes in the environment, which is primarily accomplished by two-component regulatory systems (TCSs) [72]. Bacterial TCSs comprise a sensory histidine kinase (HK) and a response regulator (RR). In response to a specific stimulus, the HK undergoes autophosphorylation, the phosphate residue is then transferred to a conserved aspartate in the RR protein. This results in a conformational change in the RR, which usually dimerizes and acts as a transcription factor [73]. TCSs and other extracellular and intracellular regulators modulate each other’s activity and alter the gene expression profile to orchestrate a cascade of reactions that promote pathogen survival [74].

The *M. tuberculosis* genome contains 12 paired TCSs, 5 single RRs and 2 unpaired sensor HKs. At least four of them (DosRST, PhoPR, MprAB and SenX3-RegX3) have been demonstrated as contributing to virulence [72]. PhoP activates the transcriptional regulator EspR, which in turn induces transcription of the espACD operon, required for ESX-1-dependent ESAT-6 secretion [75]. MprAB also modulates ESX-1 functions through the regulation of EspA transcription [76]. The DosRST regulatory system in turn (as was discussed earlier) plays a crucial role in the transition of Mtb to the resting state in response to hypoxia, nitric oxide (NO) and carbon monoxide stresses [77].

While speaking on virulence factors, one should also take into account MPT63 and MPT64. They are secretory immunodominant Mtb antigens that are highly expressed during active TB and are found in macrophages of inflammatory foci [78]. These virulence factors regulate host proteins for Mtb survival and bacterial spread in the host organism.

MPT63 has been shown to induce macrophage cell death through conformational switching in a pH-dependent manner [78]. The effect of MPT63 and MPT64 on regulating the expression of IFN-β and production of ROS was also found [79]. As well, a new role of MPT64 in stimulating the generation of myeloid-derived suppressor cells (MDSCs) that promote the survival of Mtb and avoid its destruction by the immune system has been demonstrated recently. MDSC were shown to express high levels of immunosuppressive molecules PD-L1, TIM-3, NO, arginase 1, IDO-1, IL-10 and TGF-β, but suppress the production of the pro-inflammatory cytokines TNF-α, IL-6 and IL-12. In addition, high lipid and methylglyoxal content and reduced glucose consumption put MDSCs in a metabolic quiescent state and consequently reduce the ability to phagocytize Mtb and provide a safer refuge for the intracellular survival of mycobacteria. The identified mechanism of differentiation of dendritic cells into MDSCs is based on the increased production and accumulation of methylglyoxal [80].

So, Mtb has developed a multitude of adaptations and virulence factors that serve to enhance its survival and suppress host immune responses. The secreted phosphatases PtpA, PtpB and SapM, the kinase pknG and the ESX-1 secretion system and its regulatory systems are currently considered to be the most promising targets for the development of new anti-virulence compounds [72,81,82,83]. The secreted proteins seem to be convenient targets because the drug does not need to pass through the mycobacterial cell wall, which represents a significant challenge for anti-TB drugs. The ESX-1 secretion system is also of interest as it has numerous functions associated with virulence. Inhibiting the ESX-1 system precludes the possibility of effector functioning. It should be emphasized, although many cell wall components are involved in virulence, inhibitors of mycobacterial cell wall synthesis are not considered here, as they are discussed in detail elsewhere [84].

## 3. Selection of Suitable Models for Screening and Evaluation of Virulence Factors Inhibitors

The development of new drug compounds can be approached in the following two main ways: target-to-drug and drug-to-target. The first approach involves the rational design of compounds based on the target structure followed by the evaluation of potential drug efficacy. The second approach involves the screening of extensive libraries on relevant model systems, followed by the identification of the target [85]. The drug-to-target (the second) strategy has been proven to be a highly effective method for the discovery of new antitubercular drugs and targets [8,9]. High-throughput screening (HTS), which allows for the simultaneous and rapid evaluation of hundreds or thousands of compounds, is a powerful tool for its implementation [85].

**Figure 2:**
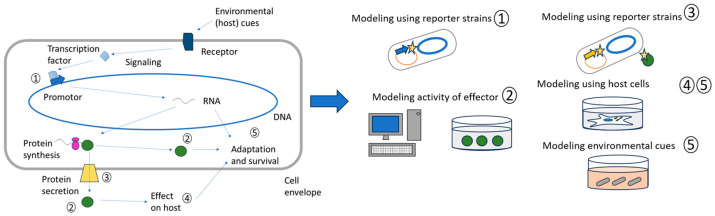
The selection of an appropriate screening model for screening virulence factor inhibitors. **Left panel**. The scheme of virulence realization represents events that can provide the output in a screening model. Once the bacterium infects the host, it is exposed to a variety of stressors. The signal from the external environment (the internal environment of the host in this case) is transmitted into bacterial cytoplasm, activates specific transcription factors and thus initiates the transcription of genes (1) that are essential for survival. Some of these genes encode effector proteins (2) that are secreted (3) across the cell membrane and/or cell wall and perform effector functions in the host (4), while some of the expressed proteins are involved in adaptation mechanisms within the bacterial cells (5). **Right panel**. The activity of regulatory systems and associated transcription factors (1) can be evaluated through the use of reporter strains, wherein the reporter gene is under the control of the promoter of the regulated region. The binding and blocking of a known reporter protein can be directly modelled in silico and screened in model systems with recombinant purified proteins (2). The activity of secretion systems can also be evaluated directly in strains where a known substrate of the secretion system is fused to a reporter (3). In vitro models using infected cells facilitate the assessment of parameters like intracellular survival of Mtb (5) and specific responses of infected cells, such as cell death (4). By in vitro simulating of aggressive conditions of the infection site and assessing the survival of Mtb cells, the function of genes responsible for adaptation and its disturbances could be evaluated.

The majority of promising tuberculosis treatments were discovered by searching for compounds that would inhibit bacterial activity in vitro [8,9]. However, this approach is usually not applicable to virulence inhibitors, as these compounds do not affect viability directly. In order to compile a suitable compound library and select an optimal model system, it is necessary to have an understanding of which processes can be blocked by the compounds (Figure 2).

The first group of methods used for drug development in a target-to-drug model are in silico methods. They have the highest throughput and the lowest labor and time costs. The virtual screening of compounds can be performed using ligand-based and structure-based techniques depending on the availability of the target’s three-dimensional structure [86]. The ligand-based drug design approach is based on known active or inactive compounds potentially interacting with the target [87]. An example of structure-based techniques is molecular docking. This method uses the virtual manipulation of the three-dimensional molecular structures of the target and the compounds under investigation for both the structural characteristics of compounds and their activities. It is also a powerful tool in the research of new anti-TB drugs [88]. Computer-aided drug design coupled with parallel and high-performance computing (HPC) platforms and new in silico machine learning-based prediction of drug–target interactions have greatly enhanced the predictive power of in silico approaches recently [89,90].

The ability of designed compounds to bind to the target must be evaluated in direct experiments. In the case of enzymes, the task is usually resolved through in vitro inhibition assays using purified recombinant proteins. A significant proportion of target-to-drug research is conducted through the evaluation of potential inhibitors on purified recombinant enzymes, frequently preceded by in silico modelling of the inhibitors binding capacity [91,92,93]. However, even if such activity is detected in model systems in vitro, the results are not always transferable to real infection conditions due to a variety of factors, including the bioavailability of the compound and its ability to penetrate mycobacterial cell wall and/or host cell membranes. Moreover, verification of the exact target of a rationally designed compound is necessary.

The most versatile screening models for the drug-to-target identification of anti-virulence compounds are based on the infection of eukaryotic cells, typically human or murine macrophages in vitro [94,95,96]. The use of multi-well plates and automated technology allows for high throughput to evaluate thousands of compounds. Such models permit direct evaluation of the effect of a compound on bacterial survival in host cells and are typically employed to verify the activity of inhibitors identified in alternative models [36,97]. Another important parameter that can be estimated in such models is the effect of mycobacteria on host cells; in the simplest case, their death [98]. The screening enables the identification of compounds with obvious cytotoxic and non-cell-penetrating properties at the early stage. In vitro models of mammalian cell infection facilitate a comprehensive evaluation of the effect of inhibitors on Mtb interactions with the host cell, providing a valuable tool for subsequent studies of detected inhibitors.

Some of the factors affecting mycobacteria during infection can also be modelled by bacteria culturing under conditions of acid stress, carbon starvation and the use of cholesterol as the main carbon source [96,99,100]. Such models reflect adaptive changes in cell metabolism and make it possible to exclude the compounds that are inactive due to decreased entry to the bacterial cell. However, the contribution of bacteria–host interactions cannot be adequately assessed in the systems.

The use of Mtb reporter strains expressing fluorescent proteins or luciferase in combination with automated fluorimetry, luminometry or microscopy methods provides a high throughput for estimating bacterial survival rates [95,101]. Other reporter strains which will be described subsequently can be utilized for identifying specific inhibitors of regulatory genes and the secretion system [102,103].

When an active substance is obtained, further optimization is usually carried out. In silico methods allow the influence to be predicted of chemical modifications of a substance on its ability to bind to the target, as well as on its physical and chemical properties that determine bioavailability [104,105]. The fragment-based drug discovery approach is based on the addition of various molecular fragments to the initial compound with a small molecular weight and low affinity to the target to finally obtain potent drug-like molecules [106].

The effect of a definite molecule on Mtb cells upon infection of macrophages allows prediction of its activity in vivo, though this does not necessarily guarantee the presence of such activity [81]. Furthermore, additional factors such as the bioavailability of the compound and the potential for accumulation of the active concentration must be considered. It is possible that Mtb may possess compensatory mechanisms that serve to escape the effects of the compound under consideration. Unfortunately, only a subset of the identified anti-virulence compounds have undergone in vivo investigations (Table 1). In the current paper, an overview is presented of the works that have identified Mtb virulence-inhibiting compounds, as was demonstrated in ex vivo infection models at least. The works to be discussed have been classified according to the putative target for which the model was selected. However, in drug-to-target studies, the precise molecular target is not always readily discernible.

## 4. Compounds Inhibiting *Mycobacterium tuberculosis* Virulence Factors

### 4.1. Cholesterol Metabolism

In an intracellular environment, Mtb employs host-derived fatty acids [110] and cholesterol [111] as a carbon source that necessitates specific metabolic adaptations. A comprehensive library of compounds was screened under in vitro infection conditions resulting in the identification of several classes of compounds that were found to impede Mtb growth. The compounds were found to block the HsaAB enzyme complex, which is required for the complete degradation of cholesterol A/B rings, as well as 2-methylcitrate synthase, that is required for the assimilation of a cholesterol-derived propionyl-CoA into the tricarboxylic acid cycle [96]. Furthermore, additional compounds were identified that target other steps in cholesterol utilization. Resistance to these compounds was determined by mutations in the adenylate cyclase Rv1625/Cya. The compounds were observed to be active in a 7H12 environment as well, where cholesterol is the primary carbon source. The catabolism of cholesterol seems to represent a pivotal pathway that is important for the adaptation of the Mtb metabolism to in vivo conditions [96].

### 4.2. Iron Transport

Mtb requires iron when persisting in the host cells and chelates its ions from the host environment with the siderophores, carboxymycobactins and mycobactins. It was shown that P-amino salicylic acid (PAS) has antitubercular effects based on the inhibition of mycobactin synthesis [112]. Studies of MbtI and MbtA enzymes that are involved in the synthesis of siderophores have led to the development of inhibitors with antimycobacterial activity [113]. Among them 5-phenylfuran-2-carboxylic acid have been identified as promising inhibitors of MbtI [113].

Proteins involved in siderophore recycling, such as the membrane components MmpS4 and MmpS5 and transporters MmpL4 and MmpL5, may be targets for inhibitors as well. Mutations in membrane proteins led to impaired recycling of siderophores and their accumulation within the bacterial cell. This accumulation of siderophores to toxic levels resulted in the bacteria self-poisoning, thus helping to reduce further Mtb virulence in the host cell [114].

### 4.3. PknG

Protein kinase G (PknG) is a member of the family of eukaryotic-like serine/threonine protein kinases. It is important for the growth of mycobacteria within the host organism [115]. The deletion of pknG was observed to facilitate the maturation of mycobacterial-containing phagosomes and reduce mycobacterial survival. In the same study, the tetrahydrobenzothiophene AX20017 was identified as an inhibitor and its activity was demonstrated in infected macrophages in vitro. It reduced intracellular mycobacterial survival but did not influence Mtb viability in the medium [36].

In addition, PknG has been demonstrated promoting autophagy induction and inhibiting autophagosome maturation during mycobacterial infection [52]. In addition, PknG appears to play a crucial role in maintaining metabolic and redox homeostasis [116]. PknG deletion has been demonstrated as impairing survival under latency conditions, including hypoxia and nutrient starvation, and to diminish resistance to antitubercular drugs [117]. Hence, it was hypothesized that PknG inhibitors may affect persistent bacteria within the granuloma and associated antibiotic resistance. Indeed, treatment with AX20017 resulted in a marked reduction in bacterial survival in latency models and the reduced survival of persistent bacteria upon antibiotic treatment of Mtb in an in vitro infection model using mouse peritoneal macrophages [118].

Overall, the Mtb genome encodes 11 putative eukaryotic-like serine/threonine protein kinases that exhibit a high degree of homology with each other and a relatively low degree of homology with their eukaryotic counterparts. On the basis, it has been predicted that molecules targeting their catalytic activity could become broad-spectrum antibiotics [119]. In particular, the compound NU-6027 was found to block both PknG and PknD enzymes, and it has been demonstrated to inhibit *M. tuberculosis* growth in both macrophage and mouse tissues. This compound was also able to inhibit the in vitro growth of mycobacteria and was initially identified in in vitro growth inhibition screening [120].

### 4.4. Secreted Phosphatases

#### 4.4.1. PtpA

During Mtb infection, the virulence factor PtpA, a member of the protein tyrosine phosphatase family, is delivered to the macrophage cytosol. It inhibits phagosome maturation through interaction with numerous eukaryotic proteins when the mycobacterium is phagocytized. PtpA influences the innate immune response, apoptosis and potentially the host lipid metabolism. One of its functions is the phosphorylation of human vacuolar protein sorting 33B (VPS33B) [51]. This protein belongs to the class C Vps, which plays a pivotal role in regulating endolysosomal trafficking [121]. Additionally, PtpA was observed to interact with the H subunit of the macrophage vacuolar H⁺-ATPase (V-ATPase), a multi-subunit protein complex which provides phagosome lumen acidification. This interaction is necessary for the dephosphorylation of VPS33B.

One of the substrates of PtpA is the human enzyme (hTFP) as well, which is a key player of lipid metabolism. A recent work [122] demonstrated that PtpA and hTFPα form a stable protein complex. The work confirmed that PtpA may be a bacterial factor that dephosphorylates hTFPα during infection, potentially affecting its mitochondrial localization or lipid β-oxidation activity during infection.

Deletion of PtpA has been shown to result in decreased Mtb survival in the macrophage-like THP1 cell line [53]. However, the knockout of the gene did not affect the bacterial load in the lungs, spleens and liver of infected mice of the C57BL/6 line, although the effect may be species-specific [123].

Several groups of inhibitors were revealed in the course of the screening of an extensive library of natural product derivatives for PtpA inhibitors. They were stevastelin, roseophilin and prodigiosins. The selected compounds were also observed inhibiting other phosphatases, including Ptp1B, which plays a pivotal role in regulating insulin receptor activity [124]. It was found that the synthetic derivatives of calchones, which are natural precursors of plant flavonoids, can inhibit PtpA activity. There were five compounds discovered with moderate and high inhibition activity (IC50 range 5–60 μM) [125]. Subsequent studies of this compounds demonstrated that one of them ((2E)-1-(2′-hydroxyphenyl)-3-(1-naphthyl)-2-propen-1-one) can inhibit the intracellular growth of Mtb while exhibiting no cytotoxicity. Additionally, it dephosphorylates the natural substrate of the mycobacterial PtpA VPS33B [126].

#### 4.4.2. PtpB

Secreted Mtb protein tyrosine phosphatase B (PtpB) displays dual activity, combining the functions of both a protein phosphatase and a phosphoinositide phosphatase [127]. The deletion of the PtpB gene results in the impaired survival of Mtb in activated, but not in resting macrophages. In a guinea pig infection model, the PtpB-deficient strain demonstrated a reduction in pathology and a decrease in bacterial load [128]. The expression of PtpB in Raw264.7 IFN-γ activated macrophages resulted in a significant increase in the survival of both H37Rv Mtb and non-pathogenic *M. smegmatis*. Furthermore, heterologous expression led to the inhibition of inflammatory cytokine expression (IL-1β, IL-6) and the inhibition of the ERK1/2 MAPK pathway (extracellular signal-regulated kinase 1/2 (ERK1/2) mitogen-activated protein kinase (MAPK) signaling). Additionally, a decreased incidence of apoptosis was observed during BCG infection [129].

In comparison to PtpA, PtpB exhibits a lesser degree of similarity to human proteins with merely 6% similarity to hPTP1B (human Ptp1B). This renders it a promising target for newly developed antitubercular drugs. Two distinct structural classes of novel PtpB inhibitors were identified. The 2-oxo-1,2-dihydrobenzo[cd]indole-6-sulfonamide and piperazinyl-thiophenyl-ethyl-oxalamide derivatives were observed blocking PtpB-mediated ERK1/2 inactivation in PtpB-overexpressing Raw264.7 cells. As well, they demonstrated the ability to suppress Mtb growth in INF-γ-induced macrophages [130].

A number of synthetic chalcones were also discovered to have activity against PtpB. They exhibited a dual inhibition of PtpA and PtpB proteins [91]. In general, this family of compounds has been identified as biologically active molecules that can work as anti-tuberculosis drugs in particular. A series of pyridyl and 2-hydroxyphenyl chalcones derivatives suppressed Mtb growth under in vitro conditions in the low micromolar concentrations [131]. In silico docking studies revealed chalcones are able to bind to the active sites of PtpA and PtpB enzymes with a higher affinity compared to previously found PtpB inhibitors. It was reported that chalone 20 ((E)-1-(pyren-1-yl)-3-(pyridin-3-yl)prop-2-en-1-one ) was predicted to bind to the Mtb PtpB through the docking. From here, it is of interest to investigate the compounds with respect to the intracellular survival of Mtb [131].

A family of compounds based on a two-site isoxazole was observed to inhibit PtpB enzyme and stimulate the reduction in the bacterial load in macrophages of the J774A.1 mouse line [132]. Further optimization of the compound through using a structure-based approach yielded a new series of compounds, one of which (substance 13) reduced bacterial survival in macrophages for both the drug-susceptible Mtb strain H37Rv and a multidrug resistant (MDR) strain. In combination with isoniazid and rifampicin, a reduction in bacterial load of up to 93% was observed. When used as monotherapy, the orally bioavailable substance 13 also resulted in a reduction in bacterial load in an acute and chronic guinea pig tuberculosis infection model [107].

Isolated from *Morus nigra* root bark, naturally occurring products that are Diels–Alder type adducts were also found to inhibit PtpB at submicromolar concentrations. One of the compounds Kuwanon G was shown to slow down Mtb growth within macrophages by 61.3% [133]. Fusarielin M isolated from the marine-derived fungus *Fusarium graminearum* effectively inhibited mycobacterial PtpB as well. The compound exhibited cellular activity in blocking PtpB-mediated extracellular signal-related kinases 1 and 2 (ERK1/2) and p38 inactivation in macrophages and substantially reduced intracellular mycobacterial growth within macrophages [134].

#### 4.4.3. SapM

The secreted acid phosphatase SapM is indispensable for the arrest of phagosome maturation. Its contribution is based on the activity towards phosphatidylinositol 3-phosphate (PI3P), which affects the localization and function of proteins containing PI3P-binding domains (FYVE, PH and PX), which are important for phagosome maturation [33,135]. The deletion of SapM leads to a significant reduction in the arrest of phagosome maturation and the suppression of intracellular bacterial growth upon infection of THP-1 macrophages as well as the attenuation of Mtb in a guinea pig model of tuberculosis infection [136].

The use of purified enzyme revealed 2-phospho-L-ascorbic acid (2P-AC) to act as a competitive inhibitor of SapM, thereby reducing the intracellular survival of Mtb in THP-1 macrophages [135]. Further screening led to the identification of three additional selective inhibitors of SapM with higher activity. All tree of this compounds contain trihydroxybenzene moiety and two of them have benzylidenemalononitrile core. An in vitro study demonstrated these compounds significantly reduced the mycobacterial load in infected human macrophages and also inhibited the intracellular growth of *Francisella tularensis* [97].

### 4.5. ESX-1 Secretion System

The Mtb genome encodes five specialized type seven secretion systems (T7SS), from ESX-1 to ESX-5 [137]. The ESX-1 and ESX-5 secretion systems are associated with virulence and can be considered as inhibitor targets.

Despite its importance, the structural complexity of the ESX-1 system poses challenges for inhibitor design, necessitating novel functional assays and structural studies. The loss of the RD1 genetic locus, which included ESX-1-encoding genes, led to the attenuation of a virulent strain of *M. bovis* and the obtaining of a vaccine strain *M. bovis* BCG. Reintroduction of the ESX-1 system of *M. bovis* or Mtb into the BCG genome restored the virulence of the vaccine strain [138]. The ESX-1 system has been shown to be required for bacterial replication at the early infection stages [139].

From the point of view of protein content, the ESX-1 secretory system consists of the transmembrane protein EccD1, three ATPases EccCa1, EccCb1 and EccA1 and the auxiliary proteins MycP1, EccB1 and EccE1 [140]. ESX-1 substrates include proteins EsxA (ESAT-6) and EsxB (CFP-10), that are encoded in the ESX-1 locus, as well as EspA, EspC and EspD, encoded in the espACD locus [141]. EspA and ESAT-6 secretion has been shown to be mutually dependent [142].

The search for potential ESX-1 inhibitors in a target-to-drug approach is quite challenging due to its insufficiently studied structure and difficult activity estimation primarily. The review by Chen et al. discusses in detail the possible classes of promising compounds based on our current knowledge of ESX-1 function [140]. For example, potential inhibitors may include molecules that inhibit ATPase activity, as ESX-1 comprises cytosolic (EccA1) and membrane-bound ATPases (EccCa1, EccCb1) [140]. Mycosin protease-1 (MycP1) could be also suggested as a molecular target. It was found that a group of compounds derived from pentapeptide boronic acid, which is an inhibitor of *M. tuberculosis* serine protease, binds to the active center of MycP1 which cleaves the ESX secretion-associated protein [143].

Currently, the main approach to finding ESX-1 inhibitors is functional tests. To detect the inhibitors of the whole ESX-1 secretion system and its main effector protein ESAT-6, a model based on the detection of lysis of human lung fibroblasts of the MRC-5 line infected with Mtb cells in vitro was used [98]. By screening, the most active compounds BTP15 and BBH7 were identified. Further investigation of the mechanism revealed that BTP15 inhibits MprB kinase that affects ESAT-6 secretion most likely through the deregulation of the espACD operon. The molecular mechanism of BBH7 activity is less clear. Presumably, it influences cell wall permeability and Mtb metal–ion homeostasis, which affects many aspects of bacterial life, including the TAT secretion pathway. Interestingly, a fibroblast model BBH7 led to a reduction in the bacterial load, while BTP15 had no such effect. On the other hand, in the model of activated THP-1 macrophages, both compounds prevented the intracellular growth of Mtb and increased the colocalization of mycobacteria with late endosome markers.

The same fibroblast lysis model was employed to identify a novel ESX-1 inhibitor, the oxadiazole compound S3 [144]. In peripheral blood human macrophages, treatment with the compound had a cytoprotective effect and led to reduced bacterial growth and promoted the increased fusion of phagosomes with lysosomes. Direct examination revealed the accumulation of secretory ESAT-6 within the cells, indicating that the secretion pathway was indeed obstructed. The general TAT secretion system remained unaltered at that. Furthermore, the substance was observed to possess dual activity, since it enhanced the efficacy of the second-line antitubercular drug ethionamide through positive regulation of the *ethA* gene, which encodes a mono-oxygenase essential for prodrug activation. The combination of low micromolar concentrations of S3 with a subinhibitory concentration of ethionamide resulted in a marked inhibition of Mtb growth. Studies with S3 derivatives have shown that ESX-1 inhibition does not appear to be associated with *ethA* regulation. Further studies are needed to decipher the mechanism of action of S3.

Since ESX-1 is a secretion system, its action can be evaluated in a model where the observed effect is substrate secretion. In this context, a reporter strain of Mtb-related microorganism *M. marinum* expressing CFP-10 fused to the luciferase gene was used to screen the library for ESX-1 inhibitor [103]. Bacteria were introduced into wells of the plate, treated with the testing compounds, and the luminescence associated with the secretion of recombinant fusion protein was evaluated in the medium. The substance 3,5-dinitrobenzamide (IMB-BZ) was found to exert a protective effect on both Mtb and *M. marinum*-infected THP-1 macrophages in vitro, and was also active in an in vivo model of chronic *M. marinum* infection of *Danio rerio* fishes. Evidence was obtained indicating that the putative mechanism of the compound action is CFP-10–EccCb1 interaction blocking. A resistance development study was also conducted. *M. marinum* was cultured in the presence of suboptimal concentrations of isoniazid, rifampicin or IMB-BZ for 2 months. The experiment resulted in the development of resistance to isoniazid and rifampicin, but not IMB-BZ.

Thus, at this point, at least four promising groups of compounds with different mechanisms of action have been found, which block ESX-1 functions in ex vivo models. The details of their mechanisms of action, the precise molecular targets as well as an analysis of the actual in vivo activity of the compounds are still to be elucidated.

### 4.6. PhoPR Two-Component System

The PhoPR system is constituted of two proteins, phoP and phoR. PhoR is responsible for the sensing of acidic pH or high salt content, and activates PhoP through phosphorylation [145]. They regulate the *aprABC* locus that responds to changes in pH [146]. As a result, PhoP-PhoR activity triggers the synthesis of genes involved in virulence and regulates the synthesis of complex lipids [147]. PhoP deletion mutants are severely attenuated, which allowed this mutation to be used for the development of Mtb-based vaccine strains [148].

PhoPR is a classical two-component signal transduction system. Phosphorylated histidine in its histidine kinase is unstable, which makes the direct screening for inhibitors of this enzyme rather difficult [98]. A fluorescent reporter strain obtained by fusing the promoter region of a PhoPR-dependent gene *aprA* upstream of green fluorescent protein (Mtb Erdman strain bearing *aprA*′::GFP and *smyc*′::mCherry fusions) was used to search for PhoP/PhoR inhibitors. Screening in an acidic medium revealed the fluorescence to be suppressed under ethoxzolamide (ETZ) usage. The study demonstrated that the suppression of the PhoPR core regulon altered the virulence-related lipid accumulation and inhibited ESX-1 secretion. The use of the inhibitor resulted in a reduced bacterial load in both bone marrow-derived mouse macrophages and in infected mice in vivo. In the latter case, the experiments were performed as follows. C57BL/6 mice were infected with the reporter Mtb strain. For 4 weeks, 5 days per week, mice in the experimental group received ETZ via oral tube. It was found under ETZ treatment that mice had a 0.72-log reduction in bacterial load in the lungs compared with mock-treated mice. Also, ETZ significantly downregulated PhoPR-dependent GFP fluorescence. The mechanism seems to be based on the inhibition of carbonic anhydrase, although its relationship to the PhoPR regulon has not yet been elucidated [108].

Another effective approach for screening was based on Foster resonance energy transfer (FRET) with PhoP labelled with Cy5 and DNA consensus motif with Cy3. Upon disruption of the Cy3–Cy5 complex, the energy transfer from Cy3 to Cy5 was aborted and fluorescence was recorded at a wavelength characteristic of the donor rather than the acceptor. The screening of 6000 compounds using this assay led to the identification of three compounds that were confirmed to directly bind PhoP to disrupt its DNA-binding activity. However, activity in cellular models has not yet been investigated [149].

### 4.7. KatG

The catalase-peroxidase KatG plays an important role in the defense of Mtb against reactive oxygen species. The found function of KatG in virulence is to catabolize peroxides generated by phagocyte NADPH-oxidase. In addition, the KatG deficient mutant was significantly attenuated in a C57BL/6 mouse infection model [37]. The role of KatG in virulence has also been shown in a guinea pig model [150].

Known inhibitors of KatG have been identified in the Mtb whole-cell screening model in the medium. It could seem surprising, but is most probably due to the specific role of KatG in defense against oxidative stress that inevitably accompanies the respiration process. The found compounds with inhibitory activity against Mtb were quinoline derivatives. The most potent of the inhibitors 2-(quinolin-4-ylmethylene) hydrazine-1-carbothioamide interfered with KatG in an in vitro model of enzyme activity. Using molecular docking, it was shown to occupy the KatG active site [151]. Another potent inhibitor, 1-(benzo[b]thiophen-2-ylmethylene) thiosemicarbazide, dismutated the activity in an in vivo model. Mtb pre-incubated with the substance formed reduced in size granulomas with a lower bacterial load when subcutaneously injected into rabbits. The substance also inhibited bacterial growth in vitro, and the ability to interact with KatG was shown using molecular docking [109].

It should be noted that the widely used anti-TB antibiotic isoniazid is a prodrug that is converted into a biologically active form by KatG [152]. From here, KatG mutations are one of the main mechanisms for the acquisition of isoniazid resistance. At the same time, while the enzyme activity is reduced, this leads to a lowered virulence, that can probably be partially compensated for by alkyl-hydroperoxidase C [153]. Since multidrug Mtb resistance implies isoniazid insusceptibility, inhibitor activities against such strains remain to be elucidated.

## 5. Conclusions

Mtb is a very successful pathogen. At all stages of its life cycle, the bacterium interferes with host cell signaling pathways to manipulate the immune response. The growing understanding of tuberculosis pathogenesis supported by genome deciphering allows for the search for new targets and drugs to overcome the Mtb infection that could not be discovered before. The combination of basic antitubercular drug therapy with the use of anti-virulence drugs seems to be an excellent approach for enhancing treatment outcomes. Virulence inhibitors may also contribute to a better understanding of the mechanisms of pathogenesis and facilitate development of new treatment strategies. In addition, the use of virulence inhibiting drugs will lead to the development of resistance becoming less likely. For Mtb, this is of particular importance, since the pathogen easily develops resistance through mutations due to long-term drug treatment rather than horizontal gene transfer.

The limited knowledge of the structural features and functional peculiarities of the new drug individual targets restricts the capability for the development of anti-virulence compounds. Nevertheless, to date, molecules that can inhibit secreted phosphatases, the ESX-1 secretion system and related regulatory mechanisms have already been discovered. For some compounds, the effects have been shown both in single-drug and adjunctive therapy with the latter being enhanced in comparison with the effects of standard antitubercular treatments. The screening systems developed to assess the functions of ESX-1 can also be used to find inhibitors and study the mechanisms of other Mtb transport and regulatory systems.

## Figures and Tables

**Table 1:** Examples of anti-virulence compounds with shown in vivo activity.

Inhibited Process	Compound	Screening Model	Effect Ex Vivo	Effect In Vivo
PtpB activity	Isoxazole derivate [107]	In vitro enzyme inhibition assay	Reduced Mtb survival in the RAW264.7 macrophage infection model	Reduction in bacterial load in a model of acute and chronic tuberculosis infection in guinea pigs
PhoPR signaling	Ethoxzolamide [108]	Screening with acidic-pH-inducible PhoPR- dependent reporter strain	Reduction in bacterial load in a model of infection of bone marrow-derived macrophages	Reduction in bacterial load in the organs of Mtb-infected mice
Secretion via ESX-1	3,5-Dinitrobenzamide IMB-BZ [103]	Screening with reporter strain *M. marinum* CFP10 + lux	Increased survival of Mtb or *M. marinum* infected macrophages	Reduction in bacterial load in an in vivo fish model of chronic infection of *M. marinum*
Inhibition KatG	1-(benzo[b]thiophen-2-ylmethylene) thiosemicarbazone [109]	In vitro anti-*Mtb* activity	-	The granulomas after treatment with compound were reduced in size, exhibited a lower bacterial load
